# Spatial Overlap of Claudin- and Phosphatidylinositol Phosphate-Binding Sites on the First PDZ Domain of Zonula Occludens 1 Studied by NMR

**DOI:** 10.3390/molecules23102465

**Published:** 2018-09-26

**Authors:** Hidekazu Hiroaki, Kaori Satomura, Natsuko Goda, Yukako Nakakura, Minami Hiranuma, Takeshi Tenno, Daizo Hamada, Takahisa Ikegami

**Affiliations:** 1 Laboratory of Structural Molecular Pharmacology, Graduate School of Pharmaceutical Sciences, Nagoya University, Furo-cho, Chikusa-ku, Nagoya, Aichi 464-8601, Japan; tenno.natsuko@f.mbox.nagoya-u.ac.jp (N.G.); nakakuray@yahoo.co.jp (Y.N.); hiranuma.minami@d.mbox.nagoya-u.ac.jp (M.H.); tenno.takeshi@e.mbox.nagoya-u.ac.jp (T.T.); 2 Division of Structural Biology, Graduate School of Medicine, Kobe University, Kusunoki-cho, Chuo-ku, Kobe 650-0017, Japan; satomura@lmls-kobe.org (K.S.); daizohamada@becellbar.co.jp (D.H.); 3 The Structural Biology Research Center and Division of Biological Science, Graduate School of Science, Nagoya University, Furo-cho, Chikusa-ku, Nagoya, Aichi 464-8601, Japan; 4 Graduate School of Engineering and Center for Applied Structural Science (CASS), Kobe University, Minatojima Minami Machi, Chuo-ku, Kobe 650-0047, Japan; 5 Institute of Protein Research, Osaka University, Suita, Osaka 565-0871, Japan; ikegamit@tsurumi.yokohama-cu.ac.jp; 6 Structural Epigenetics Laboratory, Graduate School of Medical Life Science, Yokohama-city University, Tsurumi-ku, Yokohama 230-0045 Japan

**Keywords:** phosphoinositide, protein-phospholipid interaction, PDZ domain, tight junction, NMR, chemical shift perturbation

## Abstract

*Background*: The tight junction is an intercellular adhesion complex composed of claudins (CLDs), occludin, and the scaffolding proteins zonula occludens 1 (ZO-1) and its two paralogs ZO-2 and ZO-3. ZO-1 is a multifunctional protein that contains three PSD95/Discs large/ZO-1(PDZ) domains. A key functional domain of ZO-1 is the first PDZ domain (ZO-1(PDZ1)) that recognizes the conserved C-termini of CLDs. *Methods*: In this study, we confirmed that phosphoinositides bound directly to ZO-1(PDZ1) by biochemical and solution NMR experiments. We further determined the solution structure of mouse ZO-1(PDZ1) by NMR and mapped the phosphoinositide binding site onto its molecular surface. *Results*: The phosphoinositide binding site was spatially overlapped with the CLD-binding site of ZO-1(PDZ1). Accordingly, inositol-hexaphosphate (phytic acid), an analog of the phosphoinositide head group, competed with ZO-1(PDZ)-CLD interaction. *Conclusions*: The results suggested that the PDZ domain–phosphoinositide interaction plays a regulatory role in biogenesis and homeostasis of the tight junction.

## 1. Introduction

The tight junction (TJ), or zonula occludens, is the apical-most intercellular junction complex found in epithelial and endothelial cells. TJs are responsible for the formation of functional barriers. TJs regulate the passage of cells and solutes through the paracellular space [1,2]. TJs also maintain the major barrier function of preserving the unique composition of chemical and biological substances at the apical and basolateral spaces of the cell layer. In addition, TJs are involved in some signal transduction pathways, i.e., they regulate their own assembly and barrier function, and mechanobiological signals are transmitted through TJs to the cell interior. TJs coordinate a variety of signaling and trafficking molecules that regulate cell differentiation, proliferation, and polarity; thus, TJs serve as a multifunctional complex [3,4,5].

**Figure 1:**
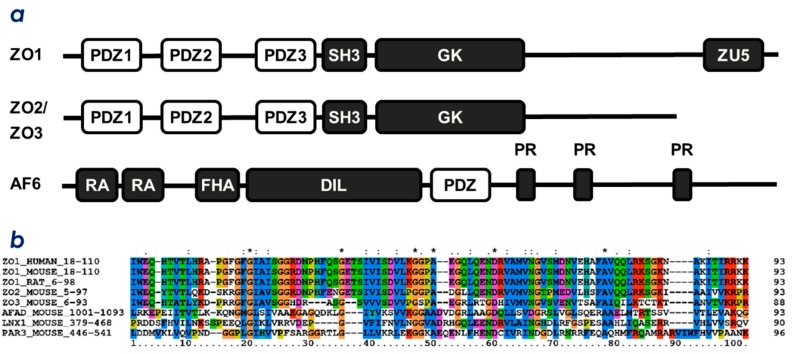
Domain architecture and multiple sequence alignment of PDZ proteins from cell-cell junctional protein complexes. (**a**) domain architectures of mouse ZO-1, ZO-2, ZO-3, and afadin (AF6). PDZ, PDZ domain; SH3, Src homology 3 domain; GK, guanylate kinase domain; ZU5, ZO-1 and UNC5 domain; RA, Ras associating domain; FHA, forkhead-associated domain; DIL, dilute domain, and PR, proline-rich region. (**b**) multiple sequence alignment of selected PDZ domains. The first PDZ domains from ZO-1/2/3 proteins, PDZ domains from afadin and Par3, and the second PDZ domain from LNX1 were aligned. Protein names and UniProtKB accession numbers are as follows: ZO-1 mouse (P39447); ZO-2 mouse (Q9WV86); ZO-3 mouse (Q9VN89); LNX1p80 mouse (Q9SEX2); afadin mouse (Q9BW62); ZO-1 human (Q07157), ZO-1 rat (F1M4A0), and Par3 mouse (Q99NH2). The sequence alignment was generated by ClustalX [11].

TJs are composed of the four integral membrane protein families: claudins (CLDs) [6], occludin [1], junctional adhesion molecules [5], and tricellulin [4]. These membrane proteins are connected to the PSD95/Discs large/ZO-1(PDZ) domain-containing proteins, such as zonula-ocludens-1 (ZO-1), ZO-2 and ZO-3. ZO-1/2/3 belong to the family of the membrane associated guanylate-kinase (MAGUK) proteins. A typical domain architecture of MAGUK proteins includes PDZ domains, a Src homology-3 (SH3) domain and a guanylate kinase-like (GUK) domain (Figure 1a) [7,8,9]. ZO proteins are postulated to tether membrane proteins at tight junctions with their multiple PDZ domains in a redundant manner [8,9]. Among them, ZO-1 is localized exclusively at tight junctions in polarized epithelia. ZO-1 is the multivalent scaffold of TJ components and promotes TJ biogenesis. Contrary, the degrading process is promoted by another PDZ-domain-containing ubiquitin E3 ligase, ligand of Numb X protein 1 p80 (LNX1p80, a splicing variant of LNX1) [10]. TJ maintenance is thought to be a rather dynamic process that is an equilibrium between biogenesis and degradation/down-regulation. Both processes (biogenesis and degradation) are regulated by proteins containing PDZ domains. 

The PDZ domain is a compact globular module, and is an interface of protein-protein interaction specific for C-terminal motifs (PDZ-binding motifs; PBM) of other proteins [12,13]. As a result, PDZ domain proteins facilitate the assembly of large protein complexes inside eukaryotic cells. Many PDZ domain proteins harbor two or more PDZ domains of different protein targets. Thus, the biological function of each PDZ domain should be characterized towards its specific binding partners as well as the context in which the domain interacts with specific cellular components. In junction complexes, the interactions between any PDZ domain from ZO proteins, LNX1, and the cytosolic C-terminal PBM of CLDs are indispensable for function [10,14]. Figure 1a illustrates the domain architecture of TJ-related multiple PDZ-domain proteins, ZO-1/2/3, and afadin. Afadin is a major scaffold protein of nectins at the adherence junction (AJ), which directly links nectins to the actin cytoskeleton to maintain stable AJs by connecting the nectin complex and the E-cadherin-catenin complex. Figure 1b also illustrates the multiple sequence alignment of the selected PDZ domains related to intercellular junctions. Among the PDZ domains listed in Figure 1a, the first PDZ domain in ZO-1 and ZO-2, ZO-1(PDZ1) and ZO-2(PDZ1), are responsible for CLD recognition [14]. This is consistent with the high content of evolutionarily conserved residues among ZO-1(PDZ1) and ZO-2(PDZ1) for several organisms (Figure 1b).

We hypothesized that TJ homeostasis is maintained by a dynamic equilibrium between ZO-1-driven promotion and LNX1-driven down-regulation. This hypothesis can explain how the total number of TJs are regulated. Nevertheless, it remains unclear how the precise position of TJs on the apicolateral membrane of the epithelial cells is regulated. Our motivation is to seek a new molecular mechanism that links TJ formation/degradation with cell polarity. In this study, we focused on phosphoinositides (PIPs) as candidates of biological substances that may regulate TJ homeostasis. It should be noted that more than 70 PDZ domains bind PIPs [15]. PDZ domains acting as PIP-binding domains is a new paradigm that was first proposed by Zimmermann [16]. Accordingly, we attempt to propose that the PDZ-PIP interactions of the junctional complex may function as a simple molecular switch to regulate TJ formation. We determined the solution structure of mouse ZO-1(PDZ1) and identified its PIP-binding site by NMR titration experiments. The PIP-binding site sterically overlaps with the CLD-binding site. Indeed, PIPs compete with the CLD C-terminal peptide for binding to ZO-1(PDZ1). The physiological consequence between this molecular switch and possible upstream pathways of TJ biogenesis regulation is discussed. 

## 2. Results

### 2.1. ZO-1(PDZ1) as a Phosphoinositide-Binding Domain

**Figure 2:**
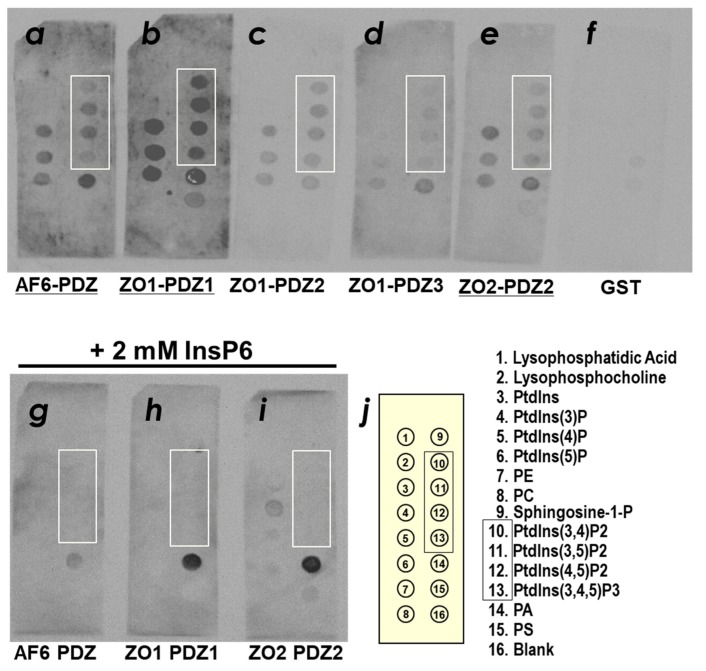
Phospholipid binding of PDZ domains among cell-cell junctional machinery proteins. Purified GST-fusion proteins of PDZ domains were examined by PIP Strip™ in the absence of phytic acid (InsP6) (**a**–**f**) and in the presence of 2 mM phytic acid (**g**–**i**). Bound proteins were detected by an anti-GST antibody. PDZ domains with marked PIP-binding activity were underlined. (**a**,**g**) mouse afadin-PDZ, (**b**,**h**) mouse ZO-1(PDZ1), (**c**) mouse ZO-1(PDZ2), (**d**) mouse ZO-1(PDZ3), (**e**,**i**) mouse ZO-2(PDZ2), and (**f**) GST alone (negative control). Spots corresponding to the phospholipid species are indicated in the box (**j**). Spots corresponding to PtsInsP_2_ and PtdInsP_3_ are in the box. PE, phosphatidylethanol; PC, phosphatidylcholine; PA, phosphatidic acid; PS, phosphatidylserine.

Several lines of evidence indicate that a certain number of PDZ domains may directly associate with lipid membranes containing PIPs [15]. Thus, we first examined the PIP-binding activity of the first PDZ domain of ZO-1, ZO-1(PDZ1), as well as several other TJ-related PDZ domains. The binding ability of the selected PDZ domains, including ZO-1(PDZ1), ZO-1(PDZ2), ZO-1(PDZ3), ZO-2(PDZ1), and afadin (PDZ) to phospholipids was examined by PIP Strip™ nitrocellulose membrane overlay assay with glutathione S-transferase (GST)-tagged PDZ domains (Figure 2). The signal intensity was quantified and summarized in Supplementary Figure S1. Among the PDZ domains examined, mouse ZO-1(PDZ1) and mouse afadin (PDZ) clearly showed binding to phospholipids. ZO-1(PDZ1) preferentially bound to phosphatidic acid (PA), phosphatidylinositol (PtdIns) mono-, bis- and triphosphates (PtdIns(3)P, PtdIns(4)P, PtdIns(5)P, PtdIns(3,4)P_2,_ PtdIns(3,5)P_2_, PtdIns(4,5)P_2_ and PtdIns(3,4,5)P_3_). Weak or no binding of ZO-1(PDZ1) to PI, PC, PE and PS was detected (Figure 2b, Supplementary Figure S1b). Similar phospholipid preference with a weaker signal intensities were observed for ZO-1(PDZ2) and ZO-2(PDZ2) (Figure 2c,e). On the other hand, afadin(PDZ) exhibited slightly different selectivity from them. ZO-1(PDZ3) did not exhibited marked PIP-binding activity. To demonstrate that these interactions are phosphoinositide head group specific, we performed the same experiment with 20 mM phytic acid (inositol hexakisphosphate). Phytic acid is a semi-specific inhibitor of various phosphoinositide-binding domains [17]. Indeed, phytic acid inhibited the binding of afadin(PDZ), ZO-1(PDZ1) and ZO-2(PDZ2) to phosphoinositides (Figure 2h). Electrostatic interactions between the positively charged residues of ZO-1(PDZ1) and the phosphate groups are likely to contribute to this binding. The interaction between PA and ZO-1(PDZ1) as well as ZO-2(PDZ2) were not inhibited by 2 mM phytic acid. 

### 2.2. Solution Structure of ZO-1(PDZ1) 

**Figure 3:**
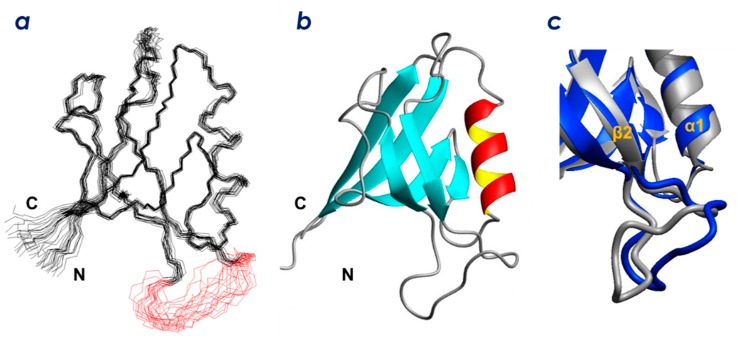
Solution structure of mouse ZO-1(PDZ1). (**a**) stereo view of the best fit superposition of the 20 structures with lowest CNS total energies. The loop residues (43–51) are shown in red. (**b**) ribbon diagram of the representative ZO-1(PDZ1) solution structure. (**c**) comparison of the solution structure (PDB: 2rrm, navy blue) and crystal structure (human ZO-1(PDZ1), PDB: 2h3m, gray).

To determine the PIP-binding site of ZO-1(PDZ1), we initially solved the structure of the domain by standard solution NMR techniques. We succeeded in assigning 90 backbone H^N^ signals out of the 95 non-Pro residues and 93% of the non-exchangeable main chain and side-chain protons and carbons [18]. We then determined the solution structure of ZO-1(PDZ1) by a combined protocol of CYANA calculations [19] followed by refinement using CNS [20]. An ensemble of 20 structures with the lowest CNS energy functions (Figure 3a) was generated from 2581 experimental NMR constraints. These 20 structures satisfy the experimental constraints (Supplementary Table S1). The stereochemical quality of the ensemble is good with all backbone phi/psi angles occupying the most favored or additionally allowed regions of the Ramachandran plot (Supplementary Figure S2). Excluding the disordered regions, i.e., the N-terminal region (residue Ile18 plus the preceding extra seven residues from the expression vector), the long loop region between β2 and β3 (residues 35–42), and the C-terminal region (residues 109–110), the r.m.s.d. values were 0.40 Å for backbone heavy atoms and 1.03 Å for all heavy atoms. 

As shown in Figure 3b, ZO-1(PDZ1) adopts a canonical PDZ domain fold. The solution structure is identical to the previously reported crystal structure of mouse ZO-1(PDZ1) in both the ligand free form (PDB ID: 2h3m) and in a complex with artificial peptide ligands (PDB: 2h2b, 2h2c) [21]. Figure 3c shows an overlay of the solution structure of ligand-free ZO-1(PDZ1) (this study, PDB: 2rrm) with the previously reported crystal structure (PDB: 2h3m). The overall backbone r.m.s.d. between the NMR structure (the representative structure, which is the closest to the average) and the crystal structure is 1.26 Å. The most remarkable structural difference is localized at the long loop between β2 and β3 (residues 35–42), where an r.m.s.d. between the NMR and X-ray structures is 5.55 Å. The loop in the NMR structure (all structures in the ensemble, shown in red in Figure 3a) adopts a relaxed shape that protrudes into the solvent. In contrast, the loop in the crystal structure adopts a specific L-shape conformation with a kink that appears to be stabilized by an electrostatic interaction between the Glu51–Arg74 side chains as well as a hydrogen bond between Ser39–His46. In the NMR structure, the loop did not converge well because structural constraints were sparse. Indeed, four of the backbone amide HSQC signals (His29, Phe30, Gln31, and Ser32) were missing in the HSQC spectra [18].

### 2.3. Determination of the CLD- and PIP-Binding Sites by NMR Titration Experiments

**Figure 4:**
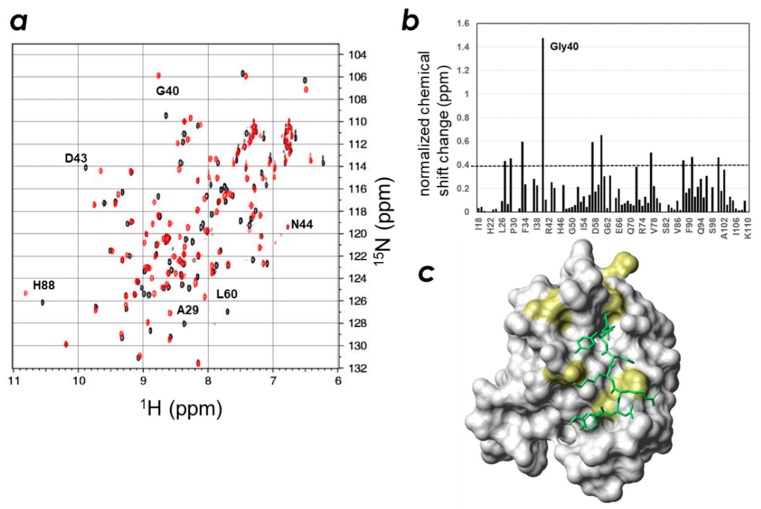
Identification of the key residues of the mouse ZO-1(PDZ1) domain responsible for CLD-3 peptide recognition. (**a**) Overlaid spectra of mouse ZO-1(PDZ1) in the presence (red) and absence (black) of the CLD-3 peptide. (**b**) Normalized chemical shift changes of mouse ZO-1(PDZ1) domain upon mixing with the 2 equivalence of the CLD-3 peptide. (**c**) Resonances representing residues with larger chemical shift changes than the threshold values are mapped onto a van der Waals surface diagram of the mouse ZO-1(PDZ1) (PDB: 2rrm) and displayed in yellow. The threshold value is indicated by the dashed line in graph (**b**).

**Figure 5:**
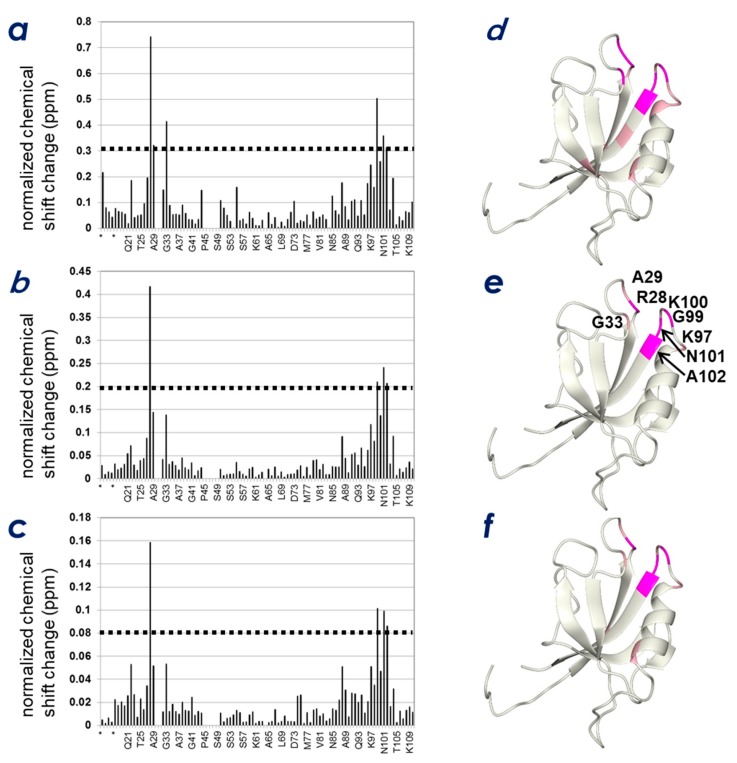
Identification of key residues of the mouse ZO-1(PDZ1) domain responsible for the interaction with inositol phosphate derivatives. Normalized chemical shift changes (**a**–**c**) and these changes mapped to the structure (**d**–**f**) upon addition of 5 molar equivalence of phytic acid (InsP6) (**a**,**d**), inositol-(1,4,5)-triphosphate, (**b**,**e**) and inositol-(1,4)-bisphosphate, (**c**,**f**). Resonances representing residues with larger chemical shift changes than the threshold values are mapped onto the ribbon model of mouse ZO-1(PDZ1) (PDB: 2rrm). The threshold values are indicated by dashed lines in the graphs. The residues at the interface are displayed in the figure.

We then titrated ZO-1(PDZ1) with the CLD-3 C-terminal peptide, Ac-GTAYDRKDYV-_COOH_, and analyzed the chemical shift changes (Figure 4, Supplementary Figure S3). We found the large signal changes for residues His27, Ala29, Gly33, Gly40, Ser57, Leu60, Met77, His88, Ala91, and Lys100, with the largest change observed for the resonance representing Gly40. These residues surround the carboxylate-recognizing residues Arg28 and Arg96 and are located at the rim of the ligand-binding groove. We then built a model structure of the ZO-1(PDZ1) + CLD-3 complex using the program Modeller (version 9.11, [22]) (Figure 4c) and the NMR results with the following caveats: (1) the C-terminal carboxylate group was recognized as the canonical PDZ-peptide interaction; and (2) the other part of the CLD peptide adopts an extended β-strand conformation. As shown in Figure 5c, the model is in good agreement with the NMR titration experiments. In addition, the suggested molecular recognition mechanism between ZO-1(PDZ1) and CLD-3 is similar to that identified by the crystal structure of the ZO-1(PDZ1)-WRRTTYL peptide complex (PDB: 2h2b), except for the additional interaction interface on the β2/β3 surface seen in the crystal structure [21]. 

Subsequently, we attempted to answer the question of the role of phosphoinositide binding in regulating ZO-1(PDZ1) activity. NMR titration experiments were performed to examine the spatial relationship between the two interfaces found on ZO-1(PDZ1); one for PIPs and the other for the CLD C-terminal peptide. As is expected for the other PIP-binding PDZ domains, we assumed that phosphatidylinositol-binding to ZO-1(PDZ1) is Ca^2+^-independent, contrary to the behavior of some other phosphoinositide-binding domains, such as the annexin, C2, and MIT domains, whose lipid binding is Ca^2+^-dependent [23,24]. Indeed, marked chemical shift changes upon titration with phytic acid, inositol-(1,4,5)-triphosphate (InsP_3_), and inositol-(4,5)-bisphosphate (InsP_2_) were observed in the absence of Ca^2+^. An example of the ^1^H-^15^N HSQC spectrum change upon phytic acid titration is shown in Supplementary Figure S4. Figure 5 shows the results of the NMR titration study with phytic acid (Figure 5a,d), InsP_3_ (Figure 5b,e), and InsP_2_ (Figure 5c,f). The ZO-1(PDZ1) structure was used to map the ligand-binding sites (Figure 5d–f). The overall profiles of the three NMR titration experiments resemble each other, except the magnitudes of chemical shift changes differ. In all cases, the inositol phosphate analogs bound the pocket (~4 Å in depth) at the edge of the peptide-binding cleft. The surface of this pocket is positively charged, where the four weakly conserved cationic residues in various PDZ domains, Arg28, Arg96, Lys97, and Lys100, contribute to form a positively charged surface of the pocket.

### 2.4. Spatial Overlap of the PIP-Binding Site and CLD C-Terminal Peptide-Binding Site on ZO-1(PDZ1) Causes Competitive Binding of Phytic Acid to the ZO-1(PDZ1)-CLD Interaction 

**Figure 6:**
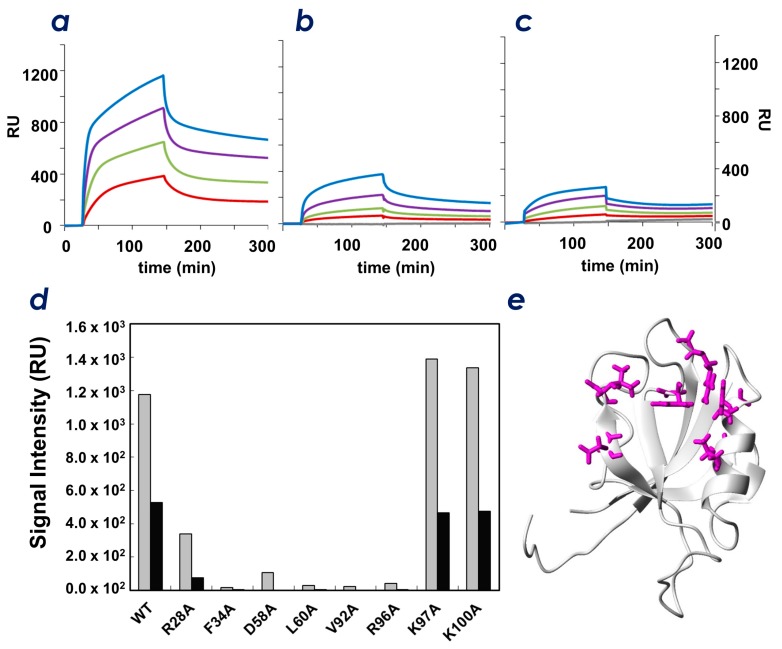
(**a**–**c**) SPR sensorgrams of the mouse ZO-1(PDZ1) with the CLD-3 peptide in the absence (**a**) and presence (**b**,**c**) of phytic acid (InsP_6_). The concentrations of phytic acid are 5 (**b**) and 10 mM (**c**). Navy lbue, magenta, green, and red lines indicate the concentration of ZO-1(PDZ1) as 100, 50, 25, and 0 μg/mL, respectively. (**d**) Signal intensities of SPR sensorgrams of mouse ZO-1(PDZ1) (wild-type and mutants) bound to the CLD-3 peptide in the absence (gray) and presence (black) of 10 mM phytic acid (InsP6). (**e**) Residues (magenta) that lose the ability to bind to CLD-3 upon alanine substitution are mapped onto a ribbon diagram of the mouse ZO-1(PDZ1) (PDB: 2rrm).

The results of the PIP-strip™ experiments (Figure 2) and the NMR titration experiments (Figure 5) indicated that ZO-1(PDZ1) directly interacts with the head groups of PIPs. In this study, we found that several residues of ZO-1(PDZ1) involved in CLD binding may also participate in PIP interaction. The results presented in Figure 4 and Figure 5 indicate that particular residues are involved in interactions with both CLD-3 peptides and PIP derivatives. For example, residue Arg28 appears to be involved in binding both moieties, whereas Lys97 and Lys100 did not contribute to CLD3-binding. This suggests that the space occupied by PIPs may spatially overlap part of the binding site occupied by the CLD-3 peptide. Thus, we examined whether the CLD peptide and PIP can bind simultaneously to the PDZ domain using phytic acid as a homolog for the PIP head group. Figure 6c shows the BIACORE™ sensorgrams recorded with CLD-3 immobilized sensor chips with ZO-1(PDZ1) as an analyte. The *K*_d_ value for CLD-3 was 170 nM. We anticipated that if the peptide and PIP-binding sites are mutually exclusive, the presence of PIP should impede the ZO-1-CLD interaction. Indeed, Figure 6a–c clearly shows that ZO-1(PDZ1) binding to the CLD-3 peptide decreases in the presence of phytic acid in a dose-dependent manner. Maximum RU values are 1200 in the absence of phytic acid, whereas it dropped to 300 in the presence of 10 mM phytic acid. This indicates that both ligands (CLDs and PIPs) are competing for the same binding site. 

### 2.5. Identification of Key Residues of ZO-1(PDZ1) That Recognize CLD

We then aimed to identify the key residues of ZO-1(PDZ1) that recognize CLD by single amino acid substitutions. From our PDZ-CLD complex model structure, we selected residues Arg28, Phe34, Asp58, Leu60, Val92, and Arg96. Residues Lys97 and Lys100 were mutated as negative controls (i.e., these two residues are not considered to be involved in ligand recognition by the domain). For the Ala-substituted mutants, the CLD-3-binding activity by SPR analysis was performed. A remarkable decrease of CLD binding activity for the Ala-substituted mutants Phe34, Asp58, Leu60, Val92, and Arg96 was observed, and a moderate decrease in binding affinity was observed for the Arg28 mutation (Figure 6a,b). Thus, Phe34, Asp58, Leu60, Val92, and Arg96 are key residues involved in binding CLD C-terminal peptides, probably through interaction with residues Val(0), Tyr(−1), and Asp(−2) at the CLD-3 C-terminus. The results indicate that the structural model of the ZO-1(PDZ1)-CLD-3 peptide complex was helpful in identifying the mechanism of molecular recognition at the residue level. 

## 3. Discussion

In this study, we found that ZO-1(PDZ1) binds to PIP_2_ and identified the binding site on this domain. In addition, the PDZ domain of afadin is a PIP-binding domain. These observations are not surprising because no less than 15% of PDZ domains were shown to bind to PIPs [16]. Moreover, the second PDZ domain (ZO-1(PDZ2)) also binds to PIP (this study and [25]). Following the discovery of the first PIP-binding PDZ domain(s) in syntenin-1 and syntenin-2 [26,27,28], structural evidence of PIP_2_ recognition by PDZ domains has accumulated [29,30]. We intended to investigate the interaction between TJ-related PDZ domains with PIPs along the context of cell-cell junction machinery biogenesis because cell adhesion and cell polarity are coupled. The increasing number of examples of PDZ-PIP interaction studies have encouraged us. In a previous study, Meerschaert et al. pointed out a weaker interaction between ZO-1(PDZ1) and PIPs than that of ZO-1(PDZ2) in a semi-quantitative manner. In this study, we succeeded in reproducing these PIP-binding interactions, ZO-1(PDZ1), ZO-1(PDZ2) and ZO-2(PDZ2) (Figure 2 and Supplementary Figure S1), but the order of affinity was different, probably due to the different sample conditions. Because ZO-1(PDZ1) plays a relevant role in tight junction biogenesis via recognizing CLDs’ C-termini, we further investigate the interplay between protein-protein interaction and protein-lipid interaction in regulating TJs. The reported binding sites of PIP_2_ for distinct PDZ domains seem varied [30]. Herein, we follow the structural classification of PIP-binding PDZ domains by Sugi et al. by classifying the various PIP-binding modes of PDZ domains into three categories [30]. We focus on the group 3 PIP-binding PDZ domains that share the same ligand-binding cleft for both canonical peptide binding and PIP-binding. Thus, the binding of a peptide ligand to PDZs should be mutually exclusive to PIP binding for all PDZ domains in this group. An example of a PDZ domain of this group is the second PDZ domain of cell-polarity modulator protein Par-3 (Par-3 (PDZ2)) [15]. In that study, the PIP-binding site of the Par-3 PDZ was shown to be overlapping with its peptide binding site. The second PDZ domains in ZO-1 and ZO-2 (ZO-1(PDZ2) and ZO-2(PDZ2)) were also reported to bind PIPs with a broad lipid specificity that belong to this group [25]. Meerschaert et al. proved that the binding of PIP_2_ competed to bind connexin 43 peptide, a peptide ligand of ZO-1(PDZ2). In the present study, we demonstrated that ZO-1(PDZ1) also belongs the group 3 PIP-binding PDZ domain that has a similar PIP and peptide-binding mode. Our results are in good agreement to the common property that PIPs may act as physiological competitors against several PDZ domain–peptide interactions. 

**Figure 7:**
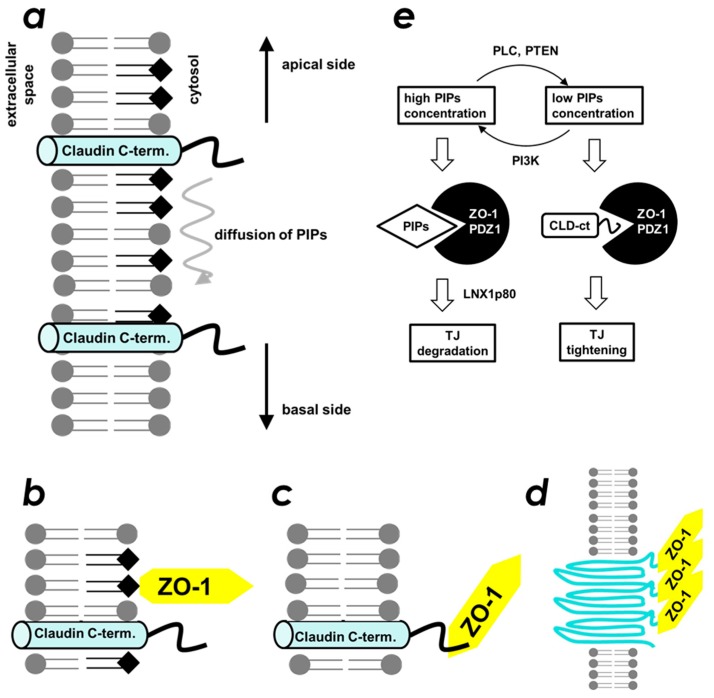
Schematic of the interplay between phosphoinositides and claudins upon ZO-1 binding. (**a**) at the apicolateral membrane, phosphoinositide distribution shows a concentration gradient from apical (top) to basal (bottom) in which the apical side (top) shows higher density. The filled squares indicate the phosphoinositide head group. The cylinder indicates the fourth helix of claudin (denoted as claudin C-term). (**b**) when high concentrations of phosphoinositides are present, ZO-1(PDZ1) binds phosphoinositides rather than the C-terminal region of claudin. (**c**) when phosphoinositide levels decrease, ZO-1(PDZ1) binds to claudins. (**d**) binding of ZO-1(PDZ1) to claudins triggers the formation of the belt-like structure of claudins. (**e**) diagram of the molecular mechanisms showing how a concentration change in phosphoinositides influences TJ biogenesis. The filled squares indicate the phosphoinositide. The cylinder with the wavy line indicates the fourth helix of claudin (denoted as CLD-ct).

The formation of the belt-like structure of TJ is believed to be important for its maintenance of barrier function. Intermolecular contacts between two neighboring CLD molecules are mainly mediated by the extracellular loops, revealed by their crystal structure [31,32,33]. Meanwhile, cytosolic interaction of ZO-1(PDZ1) and the C-terminal PBM present in CLDs is indispensable for initiation of the belt-like structure formation [2]. We hypothesized that the ZO-1-CLD interaction was regulated by PIPs by competition at the CLD-binding site of ZO-1(PDZ1). This suggests the presence of a regulatory mechanism of TJ biogenesis downstream to the PIP_2_ signaling pathway. The interplay between ZO-1-CLD and ZO-1(PDZ1)–PIP_2_ interactions may explain how cells control TJ formation in a time- and space-dependent manner. Accordingly, we propose the molecular mechanism for how the location of TJ formation is spatially regulated (Figure 7). We assume a gradient of local concentration of PIP_2_ on the apicolateral plasma membrane. For example, PIP_2_ is enriched in the apical membrane of polarized epithelial cells, whereas its concentration gradually decreases towards the basolateral membrane [34]. Accordingly, ZO-1 localizes to the plasma membrane by its first (this study) and the second [26] PDZ domains–PIP_2_ interaction. It should be noted that the ZO-1(PDZ2)-PIP_2_ interaction, which is comparable or even stronger than that of ZO-1(PDZ1). At this point, nascent CLDs are not incorporated into TJ-belts. Even when ZO-1 and CLDs are co-localized, they still do not interact with each other because ZO-1(PDZ1) is occupied by PIP_2_ (Figure 7b). Next, according to a decrease in the concentration of PIP_2_ along the top to bottom axis, ZO-1(PDZ1) may switch its binding partner from PIP_2_ to CLDs (Figure 7c). As a result, the cytosolic part of the CLDs is physically fixed, bundled, and locally concentrated. This event triggers CLDs to form an oligomer (Figure 7d). Finally, other free monomeric CLD molecules in the plasma membrane participate to extend the belt-like structure. Note that, similar interplay between afadin-nectin and afadin(PDZ)-PIP_2_ may occur, although this possibility needs further investigation. 

Our proposed mechanism is partly supported by several lines of evidence found in the literature. First, in the 1990s, the relationship between activation of phospholipase C (PLC) and TJ tightening (an increase in paracellular electric resistance) in Madin-Darby canine kidney cells was reported [35,36]. Later, inhibition of either PLCγ [37] or PLCβ [38] was reported to increase TJ permeability. Accordingly, phosphatidylinositol-3-kinase (PI3K) was also reported to regulate TJ functions. For example, in PI3Kγ-KO mice, the permeability of the blood–brain barrier (BBB) was reduced significantly [39], whereas activation of PI3K by perfluorooctane sulfonate increased the BBB permeability [40]. The results suggested that the cellular concentration of PIPs regulates TJ function. For example, enrichment of PIPs (caused by downregulation of PLC or activation of PI3K) resulted in weakened TJ function, whereas a decrease in PIPs concentration (caused by activation of PLC or inhibition of PI3K) resulted in TJ tightening (Figure 6e). We also partially succeeded in observing a similar phenomenon using a cultured MDCK II cell system. In brief, chemical inhibition of PI3K by LY-294,002 resulted in increased localization of CLD 7 at the apicolateral membrane in a dose dependent manner (Supplementary Figure S5). In addition, the connection between the PIP-specific phosphatase PTEN and the epithelial-mesenchymal transition (EMT) has been widely studied as follows. Loss or down-regulation of PTEN gene expression promotes EMT [41,42,43]. Conversely, up-regulation of PTEN via antagonizing miR-21 reverses EMT [44]. Although there are many signaling steps between PTEN and EMT, the simplest mechanism that cannot be ruled out would involve an increase in PIP concentration, which may directly loosen the TJ (Figure 7e).

## 4. Materials and Methods

### 4.1. Protein Expression and Purification

Expression vectors for the recombinant GST-tagged form of mouse ZO-1(PDZ1) (residues 18–110), mouse ZO-1(PDZ2) (residues 162–271), mouse ZO-1(PDZ3) (residues 407–512), mouse ZO-2(PDZ2) (residues 271–380), and mouse afadin(PDZ) (residues 1010–1103) were constructed using the PRESAT-vector method [45]. The fusion proteins were expressed in *Escherichia coli* BL21 (DE3), followed by affinity purification on glutathione-Sepharose FF (GE Healthcare Life Sciences Corp, Piscataway, NJ, USA) and dialyzed against the buffer containing 50 mM Tris-HCl and 150 mM NaCl (pH 7.5). The fusion proteins were used for the PIP-binding assays and SPR analysis. For NMR spectroscopy, a 2-L culture was incubated with [^15^N]-ammonium chloride and [^13^C]-glucose as the sole nitrogen and carbon sources, respectively, following a standard fermentation protocol at 20 °C. Divalent cations and vitamins were present as trace minerals and supplements during fermentation. Purification of ^15^N- and ^13^C-/^15^N-labeled ZO-1(PDZ1) was achieved by glutathione-Sepharose affinity chromatography followed by PreScission™ Protease digestion and gel filtration using a Superdex 75 column (GE Healthcare Life Sciences Corp.). Expression vectors for the recombinant His6-tagged thioredoxin (Trx) fusion protein of mouse CLD-3 C-terminus (Trx-CLD3, residues 210–219) were also constructed using the PRESAT-vector method [46]. The fusion proteins were expressed in *E. coli* BL21 (DE3), followed by affinity purification on Ni^2+^-chelating-Sepharose (GE Healthcare Life Sciences Corp.) and dialyzed against the buffer containing 50 mM Tris-HCl and 150 mM NaCl (pH 7.5). These fusion proteins were used for BIACORE™ analysis. 

### 4.2. PIP Strip Assays

To assess phospholipid-binding properties, PIP Strips™ (Echelon Bioscience Inc., Salt Lake City, UT, USA) were blocked with binding buffer containing 150 mM NaCl, 10 mM Tris-HCl, pH 7.0, and 0.05% Tween20 supplemented with 3% fatty acid-free BSA (Wako, Co. Ltd., Tokyo, Japan) for 1 h at room temperature. The strips were then incubated with purified GST fusion proteins at a concentration of 100 µg/mL in blocking buffer at room temperature for 4 h. After three washes in the binding buffer supplemented with 1% fatty acid-free BSA, PIP Strips were incubated for 16 h at 4 °C with an anti-GST antibody (Nacalai Tesque, Kyoto, Japan) in the same buffer. The strips were then incubated with a secondary antibody followed by the addition of the ECL plus reagents (GE Healthcare Life Sciences Corp.) and detected by LAS1000 (Fujifilm Co. Ltd., Tokyo, Japan).

### 4.3. NMR Spectroscopy

Samples for NMR spectroscopy contained either ^15^N- or ^13^C-/^15^N-labeled ZO-1(PDZ1) at concentrations of 0.6–0.8 mM in 5% D_2_O-95% H_2_O, 22 mM MES (pH 5.9). Backbone and side chain assignments were obtained from ^1^H-^15^N HSQC, ^1^H-^13^C HSQC, HNCA, HNCO, HN(CO)CA, HN(CA)CO, HNCACB, CBCACONH, HCC(CO)NH, CC(CO)NH, and HCCH-TOCSY spectra recorded at 25 °C using Bruker Avance (600 MHz) and Avance-III (600 MHz) spectrometers (Bruker BioSpin GmbH, Karlsruhe, Germany) equipped with cryomagnetic probes [47,48]. Data were processed using NMRPipe [49] and SPARKY [50] software. Backbone amide signals were assigned with the assistance of the program MARS [51], which has been described previously [18]. Interproton distances were obtained from three-dimensional ^13^C- and ^15^N-edited NOESY spectra recorded with a mixing time of 150 ms. Structures were calculated using a standard seven iteration protocol of the program CYANA version 2.1 [19]. All nuclear Overhauser effect (NOE) cross peaks were selected manually using SPARKY and then assigned by CYANA. In total, 2427 NOE upper distance restraints were obtained, including 1023 long-range distances (Supplementary Table S1). Dihedral angle restraints were calculated on the basis of backbone nuclei chemical shifts using the TALOS program [52]. No distance restraint was violated by more than 0.3 Å and no torsional restraint by more than 5.0° during CYANA calculation. A final refinement process was performed by CNS 1.2 [20]. The 20 structures with the lowest restraint energies were selected and analyzed using MOLMOL [53] and PROCHECK-NMR software [54] (Supplementary Table S1). All figures were prepared using MOLMOL and PyMOL (http://www.pymol.org). The atomic coordinates of the 20 best ZO-1(PDZ1) NMR structures have been deposited in the Protein Data Bank under accession code 2rrm (http://www.pdbj.org/). Chemical shift assignments have been deposited in the BioMagResBank under accession code 11424 (http://www.bmrb.wisc. edu/).

### 4.4. NMR Titration Experiments

To examine the interface of ZO-1(PDZ1) with phosphoinositide analogs and the CLD-3 derived peptide, a series of 2D ^1^H-^15^N HSQC spectra with WATERGATE water suppression [55] were recorded at 30 °C, in 5% D_2_O-95% H_2_O containing 22 mM MES buffer (pH 5.8). In the titration, 0 and 5 molar equivalence of sodium salt of phytic acid, inositol-(1,4,5)-triphosphate, and inositol-(1,4)-bisphosphate were added to 0.1 mM ^15^N-labeled ZO-1(PDZ1). Mother liquid of sodium phytic acid for the titration study was prepared by diluting 50% phytic acid solution (approximate 1.1 M) and adjusted to pH 5.8 by adding an aliquot of 1 M NaOH. Similarly, 0, 1, and 2 molar equivalence of chemically synthesized, HPLC-purified CLD3-peptide (Ac-GTAYDRKDYV-_COOH_) were added to 0.1 mM ^15^N-labeled ZO-1(PDZ1). All normalized chemical shift changes in the ^1^H-^15^N HSQC spectra upon ligand titration were calculated as Δδ_normalized_ = {Δδ(^1^H)^2^ + [Δδ(^15^N)/5]^2^}^1/2^, where Δδ(^1^H) and Δδ(^15^N) are chemical shift changes in amide proton and amide nitrogen, respectively.

### 4.5. Surface Plasmon Resonance (SPR) Analysis

The binding of PDZ domains to the CLD-3 C-terminal peptide and the effect of phytic acid were examined by SPR using a BIACORE™-3000 instrument (GE Healthcare Life Sciences Corp.). The equilibrium-binding affinity of the Trx-CLD3 peptide immobilized on a CM5 chip was analyzed by monitoring the change in response units as a function of PDZ domain concentration (ranging from 0 to 100 μg/mL) at a flow rate of 20 μL/min in buffer containing 10 mM HEPES, pH 7.4, 150 mM NaCl, 3 mM EDTA, and 0.005% Surfactant P20. The *K*_D_ value was calculated by the “Affinity from steady-state” method according to the manufacturer’s instruction. For competition assay, 0, 5, or 10 mM of phytic acid was pre-incubated with ZO-1(PDZ) as the analyte. The same buffer as above was supplemented with the same concentration (0, 5, or 10 mM) of phytic acid.

### 4.6. Mutation Studies and CLD-3 Binding Assays

Ala-substituted mutants were prepared by PCR amplification of the entire expression plasmid for ZO-1(PDZ1), according to a standard PCR mutagenesis method using the QuikChange™ site-directed mutagenesis kit (Stratagene, La Jolla, CA, USA). Two complementary oligonucleotides with mutated sequences for each mutant were used as primers (Supplementary Table S2). The resulting ZO-1(PDZ1) genes were sequenced to confirm the mutations. All proteins were purified with glutathione-Sepharose (GE Healthcare Life Sciences Corp.) and dialyzed against a buffer containing 50 mM Tris-HCl and 150 mM NaCl (pH 7.5).

## 5. Conclusions

We found that ZO-1(PDZ1) exhibits PIP-binding activity. The PIP-binding site spatially overlaps with the canonical peptide-binding site of ZO-1(PDZ1). We confirmed that the domain binds to either a PIP molecule or a CLD peptide in a mutually exclusive manner. This feature allowed us to propose a model that describes the spatial regulation of TJ formation in the cell (Figure 7).

## Supplementary Materials

The following are available online, Figure S1: Quantitative analysis of phospholipid binding of PDZ domains among cell-cell junctional machinery proteins, Figure S2: Ramachandran plot for 20 Structures of ZO-1(PDZ1), Figure S3: Overlaid spectra of mouse ZO-1(PDZ1) upon titration of the indicated equivalence of claudin-3 peptide. 0 eq (black), 1 eq (navy blue) and 2 eq (red), Figure S4: overlaid spectra of mouse ZO-1(PDZ1) in the presence (orange) and absence (black) of 5 eq of phytic acid, Figure S5: Effects of PI3K inhibitor LY-294,002 on tight junction integrity of MDCK II cells. Table S1: Experimental Restraints and Statistics for 20 Structures of ZO-1(PDZ1), Table S2: DNA Primers Used for Generating Ala-substituted Mutants of ZO-1(PDZ1).
