# Supplementary material for: Spatial Overlap of Claudin- and Phosphatidylinositol Phosphate-Binding Sites on the First PDZ Domain of Zonula Occludens 1 Studied by NMR
Source: Molecules. 2018 Sep 26;23(10):2465. doi: 10.3390/molecules23102465 (PMC6222848; doi:10.3390/molecules23102465)
Supplement: Supplementary file 1 [file molecules-23-02465-s001.pdf]

Supplementary materials.

Spatial overlap of claudin- and phosphatidylinositol phosphate-binding sites on the first PDZ domain of zonula occludens 1 studied by NMR

Hidekazu Hiroaki [1,2,3,*], Kaori Satomura [2], Natsuko Goda [1,2], Yukako Nakakura [1], Minami Hiranuma [1], Takeshi Tenno [1,2], Daizo Hamada [2,4] , Takahisa Ikegami [5,6]

[1] Laboratory of Structural Molecular Pharmacology, Graduate School of Pharmaceutical Sciences, Nagoya University, Furo-cho, Chikusa-ku, Nagoya, Aichi 464-8601, Japan; tenno.natsuko@f.mbox.nagoya-u.ac.jp (N.G.); nakakuray@yahoo.co.jp (Y.N.), tenno.takeshi@e.mbox.nagoya-u.ac.jp (T.T.)

[2] Division of Structural Biology, Graduate School of Medicine, Kobe University, Kusunoki-cho, Chuo-ku, Kobe 650-0017, Japan; satomura@lmls-kobe.org (K.S.)

[3] The Structural Biology Research Center and Division of Biological Science, Graduate School of Science, Nagoya University, Furo-cho, Chikusa-ku, Nagoya, Aichi 464-8601, Japan

[4] Graduate School of Engineering and Center for Applied Structural Science (CASS), Kobe University, Minatojima Minami Machi, Chuo-ku, Kobe 650-0047, Japan; daizohamada@becellbar.co.jp (D.H.)

[5] Institute of Protein Research, Osaka University, Suita, Osaka 565-0871, Japan

[6] Structural Epigenetics Laboratory, Graduate School of Medical Life Science, Yokohama-city University, Tsurumi-ku, Yokohama 230-0045 Japan; ikegamit@tsurumi.yokohama-cu.ac.jp (T.I.)

* Correspondence: hiroaki.hidekazu@f.mbox.nagoya-u.ac.jp; Tel.: +81-52-789-4535

Supplementary Figure S1 (related to Figure 2)
Quantitative analysis of phospholipid binding of PDZ domains among cell-cell junctional machinery proteins. Results of phospholipid nitrocellulose membrane overlay assay shown in Figure 2 (GST-tagged PDZ domains examined by PIP Strip™ in the absence of phytic acid ($InsP_6$) (a–f) and in the presence of 2 mM phytic acid (g, h, and i)) were quantified by image analysis. The signal intensity was quantified by software ImageJ. After the background signal was subtracted, normalized signal intensities (the strongest spot in Figure 2, panel i is set to 100%) were indicated. (a, g) mouse afadin-PDZ, (b, h) mouse ZO-1(PDZ1), (c) mouse ZO-1(PDZ2), (d) mouse ZO-1(PDZ3), (e, i) mouse ZO-2(PDZ2), and (f) GST alone (negative control). LPA, lysophosphatidic acid; LPC, lysophosphatidylcholine; PI, phosphatidylinositol; P3, phosphatidylinositol-3-monophosphate; P4, phosphatidylinositol-4-monophosphate; P5, phosphatidylinositol-5-monophosphate; PE, phosphatidylethanol; PC, phosphatidylcholine; S1P, sphingosine-1-phosphate; P34, phosphatidylinositol-3,4-bisphosphate; P35, phosphatidylinositol-3,5-bisphosphate; P45, phosphatidylinositol-4,5-bisphosphate; P345, phosphatidylinositol-3,4,5-triphosphate; PA, phosphatidic acid; PS, phosphatidylserine.

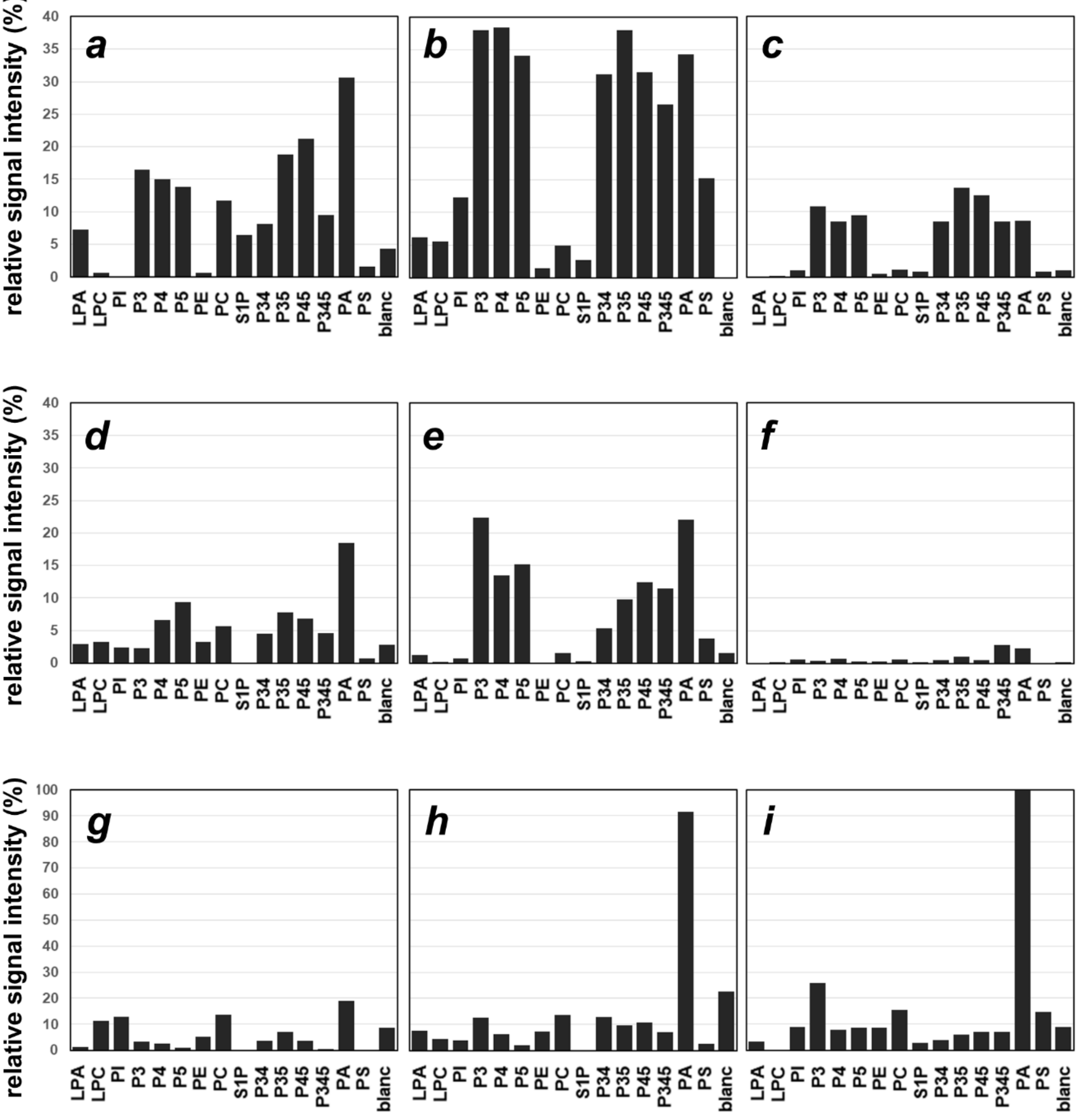

Supplementary Figure S2

Ramachandran plot for 20 Structures of ZO-1(PDZ1)

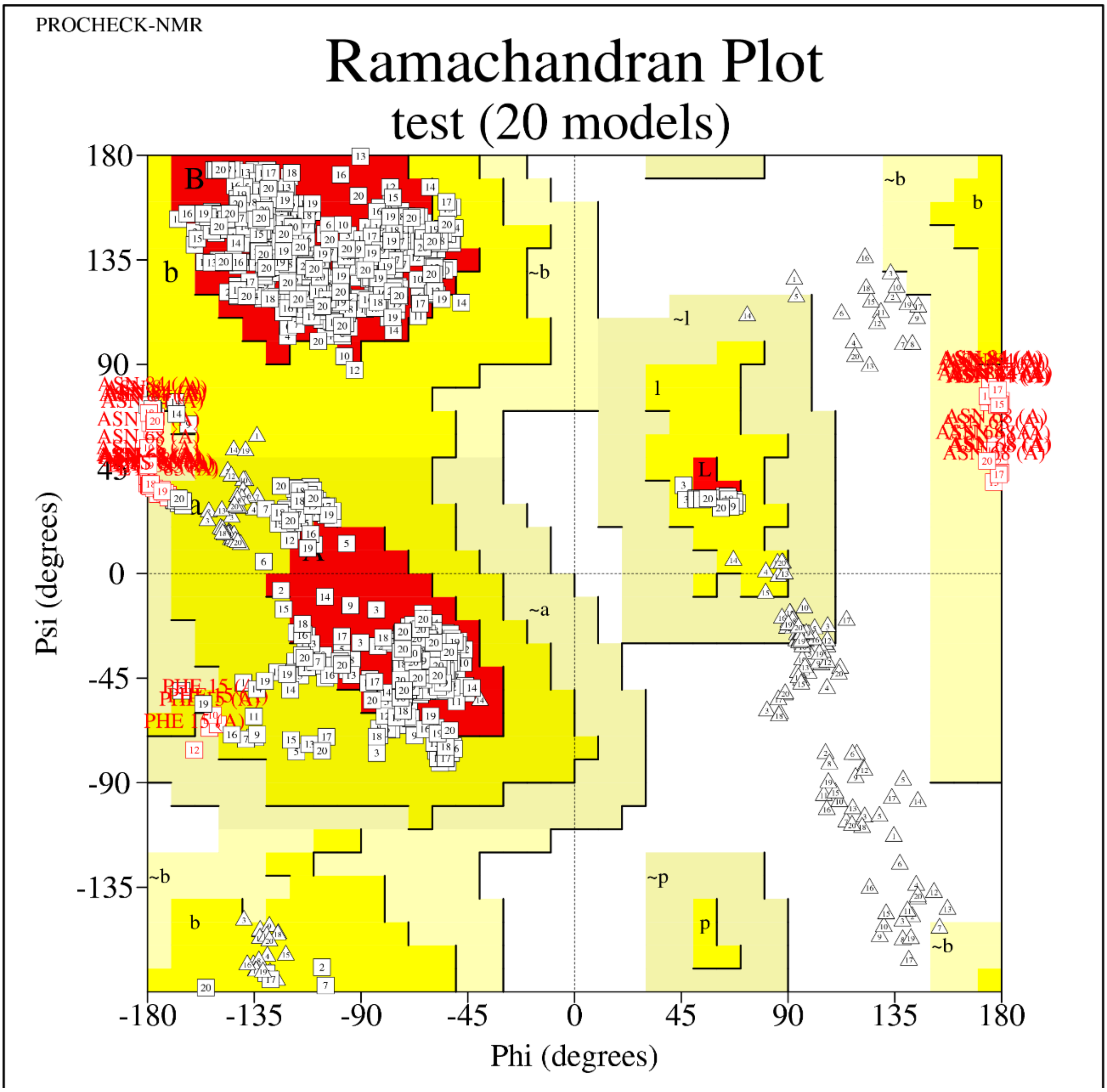

Supplementary Figure S3 (related to Figure 4)

Overlaid spectra of mouse ZO-1(PDZ1) upon titration of the indicated equivalence of claudin-3 peptide. 0 eq (black), 1 eq (navy blue) and 2 eq (red). With 1 eq (0.2 mM) of CLD3, the signal changes were mostly saturated.

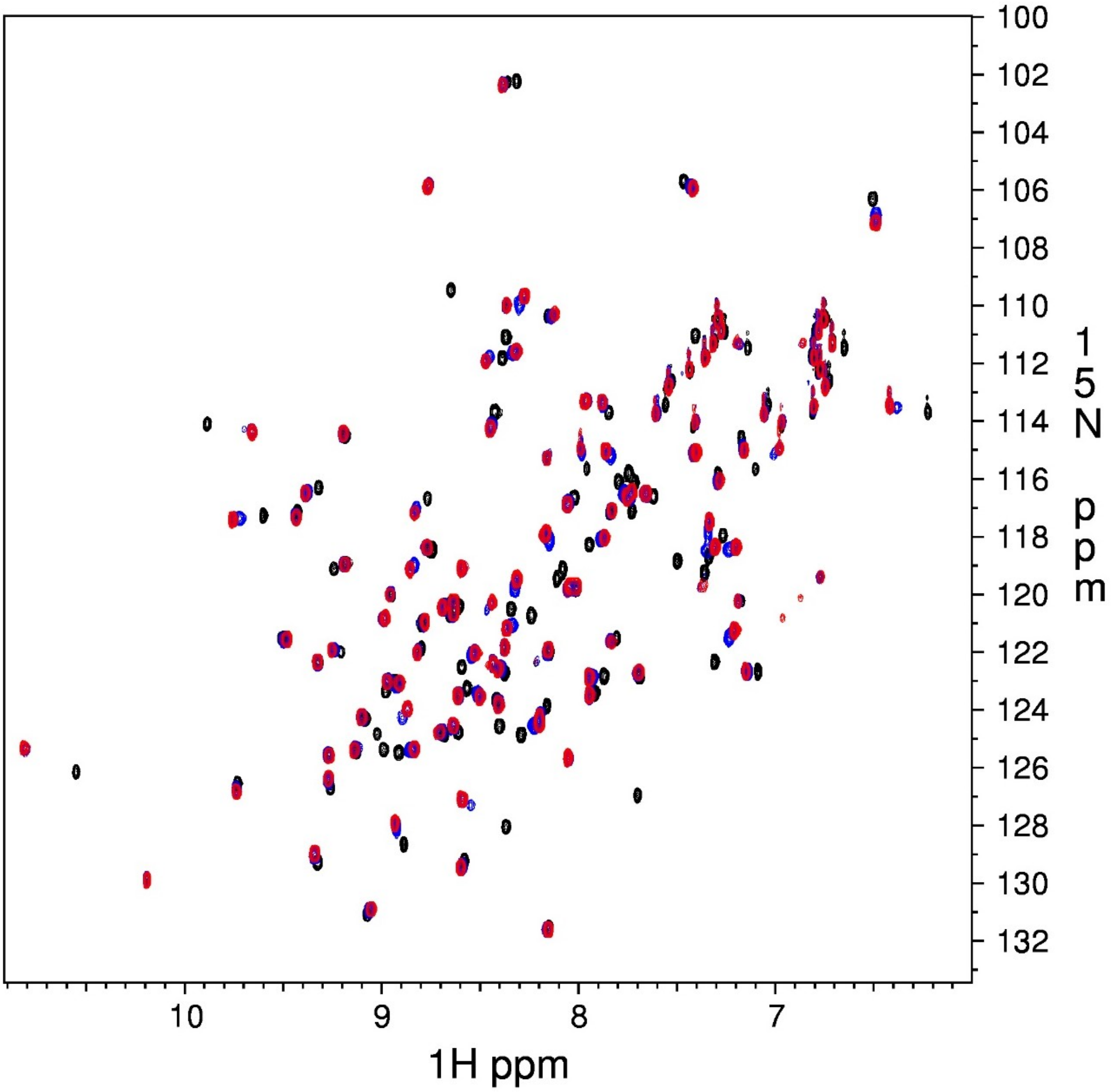

Supplementary Figure S4 (related to Figure 5)
Overlaid spectra of mouse ZO-1(PDZ1) in the presence (orange) and absence (black) of the 5 eq. of phytic acid.

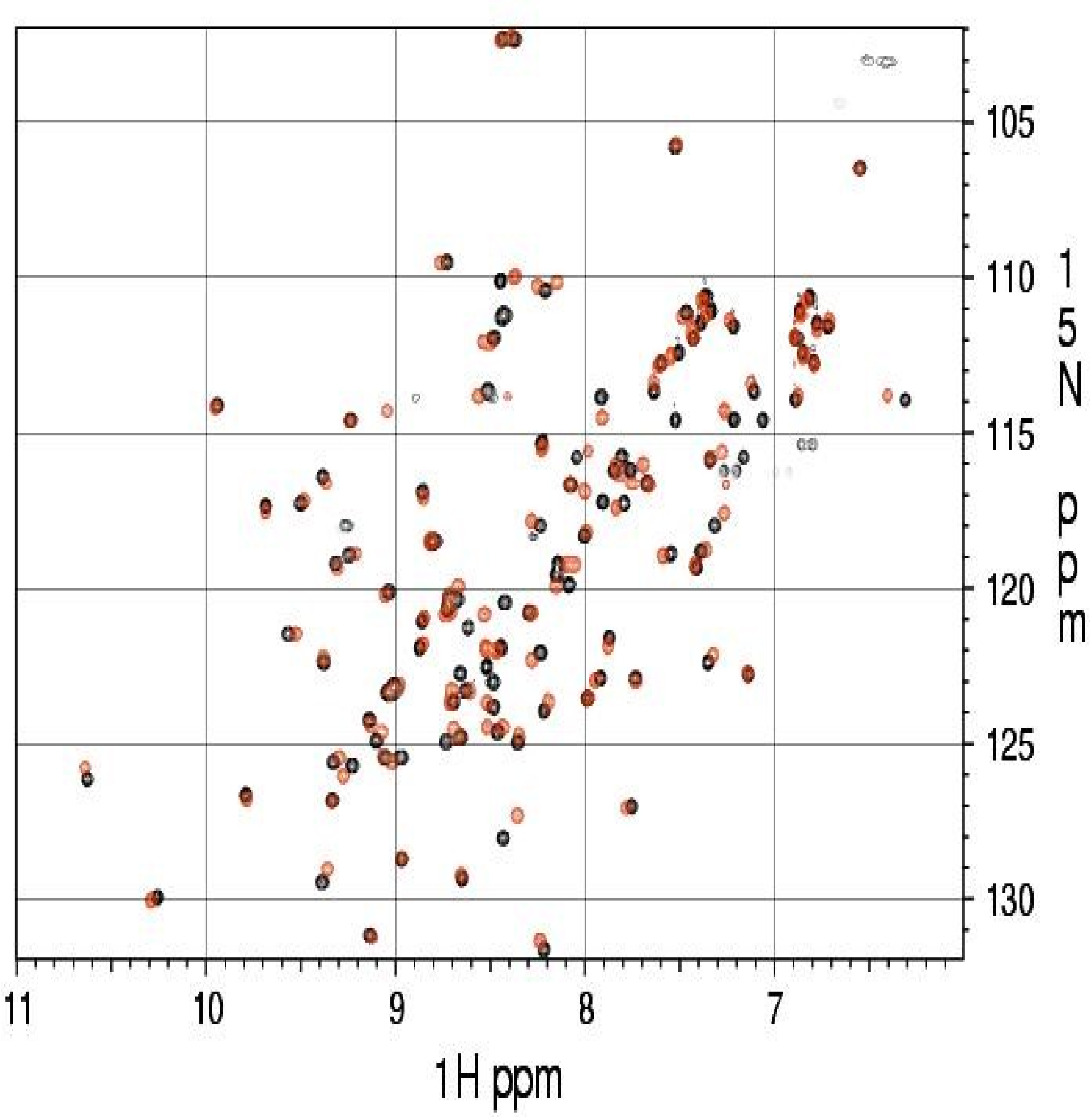

Supplementary Figure S5 (related to Figure 7)
Effects of PI3K inhibitor LY-294,002 on tight junction integrity of MDCK II cells. Apicolateral membrane localization of claudin 7 in MDCK II cell increased in a dose dependent manner of LY-294,002. *a,* 0 μM, *b*, 5 μM, *c*, 15 μM, *d*, 25 μM. For each condition, two different views from the same cultured well were shown.

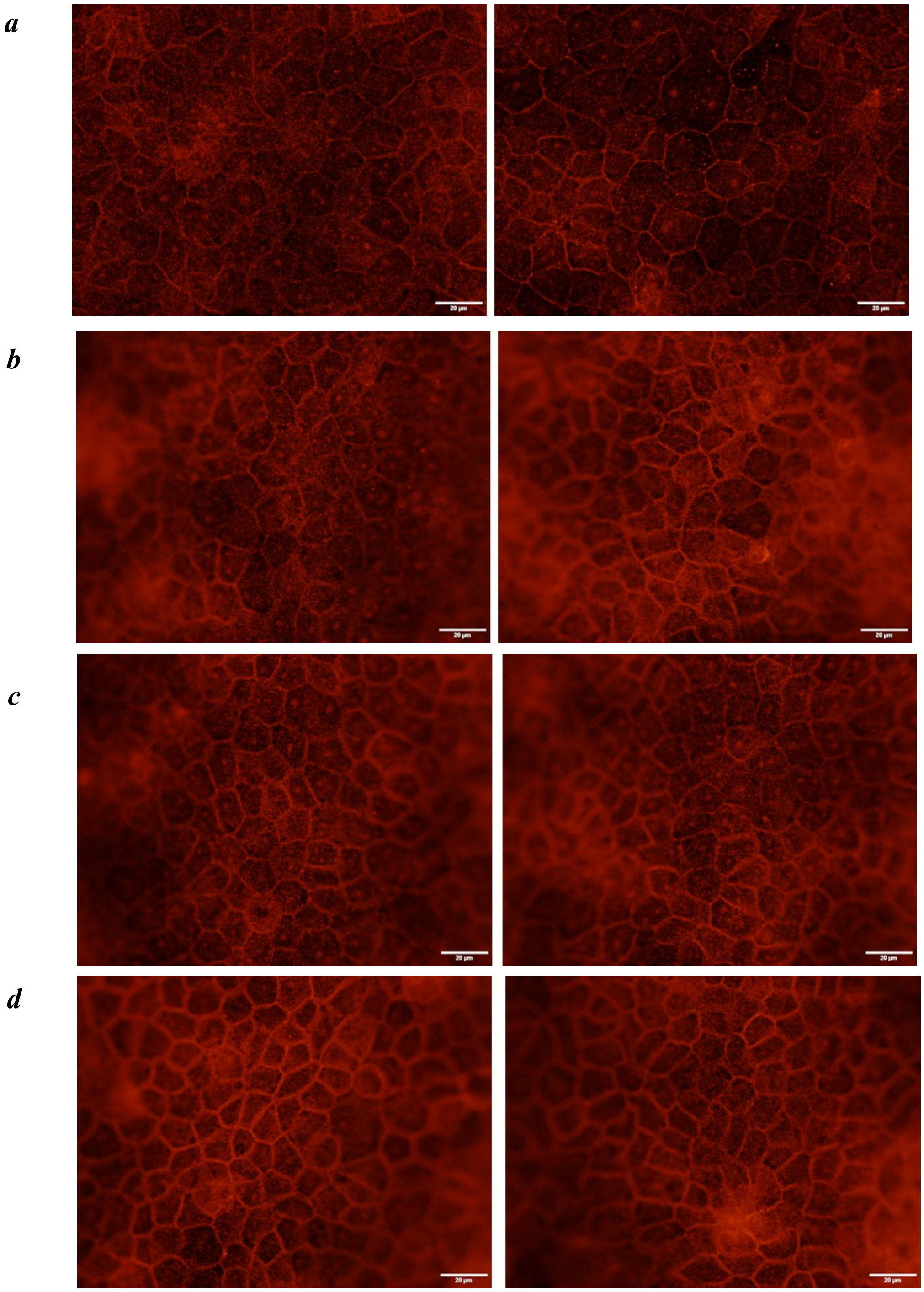

Experimental Section for Supplementary Figure S5.

MDCK II cells were cultured in Dulbecco's modified Eagle's medium (DMEM) supplemented with 10% fetal calf serum containing penicillin and streptomycin at 37 °C with 5% CO_2. Aliquots of 3.0 x 10^4 cells were plated on each well of a 6-well x 35 mm plate (Corning), and the culture medium was changed each every 2 days. 5 day after plated, the culture medium was changed to the same medium containing the indicated concentration of LY-294,002. After 48 hours of exposure to the inhibitor, the cells were fixed with cold 1 x phosphate buffered saline containing 4 % paraformaldehyde and incubated with primary antibodies for 24 hours at 4 °C. The cells were then incubated with secondary antibodies for 1 hour at room temperature. The fluorescence images were obtained using fluorescence microscopy equipped with a color CCD camera (IX-71 and DP-70, respectively, Olympus, Japan). Scale bar: 20 µm.

Supplementary Table S1.

Experimental Restraints and Statistics for 20 Structures of ZO-1(PDZ1)

**Distance Restraints**	
Total number of restrains	2428
Intra-residual	324
Sequential restraints [| i-j | = 1]	646
Medium-range restraints [1 < | i-j | ≤ 4]	435
Long-range restraints [| i-j | > 4]	1023
Dihedral angle restraints	109
Phi/Psi/Chi	54/55/0
Hydrogen bond restraints	22 x 2
**Statistics used for and obtained from the Structure Calculations**	
Final Statistics (20/50)	
Cutoffs: Distance (0.3 Å) and Angle (3.0 deg.)	
Maximum CYANA target function	4.85
Maximum violations	
Distance violation (Å)	0.67
Angle violation (deg.)	15.80
Maximum CNS Overall Energy	37.03
Coordinate precision (residues 19-42,52-107)*	
Backbone rmsd. (Å)	0.40
Heavy atoms rmsd. (Å)	1.03
Ramachandran plot statistics (%) (residues 19-42,52-107)*	
Residues in most favored regions	85.6
Residues in additionally allowed regions	11.0
Residues in generously allowed regions	3.4
Residues in disallowed regions	0.0

* Residue numbers are correspond to those in the full length mouse ZO-1 (accession:NP_033412)

Supplementary Table S2.

DNA primers used for generating Ala-substituted mutants of ZO-1(PDZ1)

**R28A**	forward	gtgacgcttcacgcggctcctgggtttgg
	reverse	ccaaacccaggagccgcgtgaagcgtcac
**F34A**	forward	ggctcctgggtttggagctggaattgcaatctctg
	reverse	cagagattgcaattccagctccaaacccaggagcc
**D58A**	forward	cctccatagtgatttctgcagtgttaaaaggagggccag
	reverse	ctggccctccttttaacactgcagaaatcactatggagg
**L60A**	forward	gtgatttctgatgtggcaaaaggagggccagctgaagg
	reverse	ccttcagctggccctccttttgccacatcagaaatcac
**V92A**	forward	gaacatgcttttgctgctcagcagctaaggaagagtgg
	reverse	ccactcttccttagctgctgagcagcaaaagcatgttc
**R96A**	forward	gctgttcagcagctagcgaagagtgggaaaaacgc
	reverse	gcgtttttcccactcttcgctagctgctgaacagc
**K97A**	forward	gctgttcagcagctaagggcgagtgggaaaaacgc
	reverse	gcgtttttcccactcgcccttagctgctgaacagc
**K100A**	forward	gctaaggaagagtggggcaaacgcaaaaattactatccg
	reverse	cggatagtaatttttgcgtttgccccactcttccttagc
